# Normalized Metadata Generation for Human Retrieval Using Multiple Video Surveillance Cameras

**DOI:** 10.3390/s16070963

**Published:** 2016-06-24

**Authors:** Jaehoon Jung, Inhye Yoon, Seungwon Lee, Joonki Paik

**Affiliations:** 1 Department of Image, Chung-Ang University, 84 Heukseok-ro, Dongjak-gu, Seoul 06974, Korea; gjslkjs@gmail.com (J.J.); inhyey@gmail.com (I.Y.); superlsw@gmail.com (S.L.); 2 ADAS Camera Team, LG Electronics, 322 Gyeongmyeong-daero, Seo-gu, Incheon 22744, Korea; 3 Software Development Team, Convergence R&D Center, LG Innotek, Gyeonggi-do 15588, Korea

**Keywords:** video surveillance, video retrieval, automatic calibration, metadata descriptor, homology, color clustering, object tracking

## Abstract

Since it is impossible for surveillance personnel to keep monitoring videos from a multiple camera-based surveillance system, an efficient technique is needed to help recognize important situations by retrieving the metadata of an object-of-interest. In a multiple camera-based surveillance system, an object detected in a camera has a different shape in another camera, which is a critical issue of wide-range, real-time surveillance systems. In order to address the problem, this paper presents an object retrieval method by extracting the normalized metadata of an object-of-interest from multiple, heterogeneous cameras. The proposed metadata generation algorithm consists of three steps: (i) generation of a three-dimensional (3D) human model; (ii) human object-based automatic scene calibration; and (iii) metadata generation. More specifically, an appropriately-generated 3D human model provides the foot-to-head direction information that is used as the input of the automatic calibration of each camera. The normalized object information is used to retrieve an object-of-interest in a wide-range, multiple-camera surveillance system in the form of metadata. Experimental results show that the 3D human model matches the ground truth, and automatic calibration-based normalization of metadata enables a successful retrieval and tracking of a human object in the multiple-camera video surveillance system.

## 1. Introduction

Multiple camera-based video surveillance systems are producing a huge amount of data every day. In order to retrieve meaningful information from the large data set, normalized metadata should be extracted to identify and track an object-of-interest acquired by multiple, heterogeneous cameras.

Hampapur et al. proposed a real-time video search system using video parsing, metadata descriptors and the corresponding query mechanism [1]. Yuk et al. proposed an object-based video indexing and retrieval system based on object features’ similarity using motion segmentation [2]. Hu et al. proposed a video retrieval method for semantic-based surveillance by tracking clusters under a hierarchical framework [3]. Hu’s retrieval method works with various queries, such as keywords-based, multiple object and sketch-based queries. Le et al. combined recognized video contents with visual words for surveillance video indexing and retrieval [4]. Ma et al. presented a multiple-trajectory indexing and retrieval system using multilinear algebraic structures in a reduced-dimensional space [5]. Choe et al. proposed a robust retrieval and fast searching method based on a spatio-temporal graph, sub-graph indexing and Hadoop implementation [6]. Thornton et al. extended an existing indexing algorithm in crowded scenes using face-level information [7]. Ge et al. detected and tracked multiple pedestrians using sociological models to generate the trajectory data for video feature indexing [8]. Yun et al. presented a visual surveillance briefing system based on event features, such as object’s appearances and motion patterns [9]. Geronimo et al. proposed an unsupervised video retrieval system by detecting pedestrian features in various scenes based on human action and appearance [10]. Lai et al. retrieved a desired object using the trajectory and appearance in the input video [11]. The common challenge of existing video indexing and retrieval methods is to summarize infrequent events from a large dataset generated using multiple, heterogeneous cameras. Furthermore, the lack of normalized object information during the search prevents from accurately identifying the same objects acquired from different views.

In order to solve the common problems of existing video retrieval methods, this paper presents a normalized metadata generation method from a very wide-range surveillance system to retrieve an object-of-interest. For automatic scene calibration, a three-dimensional (3D) human model is first generated using multiple ellipsoids. Foot-to-head information from the 3D model is used to estimate the internal and external parameters of the camera. Normalized metadata of the object are generated using the camera parameters of multiple cameras. As a result, the proposed method needs neither a special calibration pattern nor a priori depth measurement. The stored metadata can be retrieved using a query, such as size, color, aspect ratio, moving speed and direction.

This paper is organized as follows. Section 2 describes the 3D human model using multiple ellipsoids. A human model-based automatic calibration algorithm and the corresponding metadata retrieval method are respectively presented in Section 3 and Section 4. Section 5 summarizes the experimental results, and Section 6 concludes the paper.

## 2. Modeling Human Body Using Three Ellipsoids

**Figure 1:**
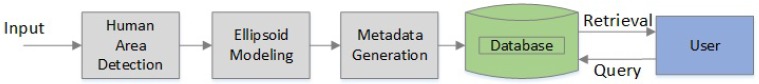
Block diagram of the proposed human retrieval method.

A multiple camera-based surveillance system must be able to retrieve the same object in different scenes using an appropriate query. However, non-normalized object information results in retrieval errors. In order to normalize the object information, we estimate camera parameters using automatic scene calibration and then estimate a projective matrix using camera parameters obtained by scene calibration. After obtaining normalized information, the object in the two-dimensional (2D) image is projected to a 3D world coordinate using the projection matrix. Existing camera calibration methods commonly use a special calibration pattern [12], which extracts feature points from a planar pattern board and then estimates the camera parameters using a closed-form solution. However, the special calibration pattern-based algorithm has a limitation because the manual calibration of multiple cameras at the same time is impractical and inaccurate. In order to solve this problem, we present a multiple ellipsoid-based 3D human model using the perspective property of 2D images, and the block diagram of the proposed method is shown in Figure 1.

Let $X_{f}={\left[X_{f}\;Y_{f}\;1\right]}^{T}$ be the foot position on the ground plane and $x_{f}={\left[x_{f}\;y_{f}\;1\right]}^{T}$ the corresponding foot position in the image plane, all in the homogeneous coordinate. Given $x_{f}$, $X_{f}$ can be computed using the homography as:

(1) $$X_{f}=H^{-1}x_{f}$$

where $H={\left[p_{1}\;p_{2}\;p_{3}\right]}^{T}$ is the 3×3 homography matrix, and $p_{i}$ for i=1,2,3 are the first three columns of the 3×4 projection matrix *P* that is computed by estimating camera parameters. We then generate the human model with height *h* on the foot position using three ellipsoids, including head $Q_{h}$, torso $Q_{t}$ and leg $Q_{l}$, in the 3D world coordinate. The 4×4 matrix of the ellipsoid is defined as [13]:

(2) $$Q_{k}=\left[\begin{array}{cccc} \frac{1}{R_{X}^{2}} & 0 & 0 & -\frac{X_{c}}{R_{X}^{2}}\\ 0 & \frac{1}{R_{Y}^{2}} & 0 & -\frac{Y_{c}}{R_{Y}^{2}}\\ 0 & 0 & \frac{1}{R_{Z}^{2}} & -\frac{Z_{c}}{R_{Z}^{2}}\\ -\frac{X_{c}}{R_{X}^{2}} & -\frac{Y_{c}}{R_{Y}^{2}} & -\frac{Z_{c}}{R_{Z}^{2}} & \frac{X_{c}^{2}}{R_{X}^{2}}+\frac{Y_{c}^{2}}{R_{Y}^{2}}+\frac{Z_{c}^{2}}{R_{Z}^{2}} \end{array}\right]$$

where $Q_{k}$, k∈{h,t,l}, respectively, represent the ellipsoid matrices of head, torso and leg. $R_{X}$, $R_{Y}$ and $R_{Z}$ respectively represent the radii of ellipsoids in *X*, *Y* and *Z* coordinates and ${\left[X_{c}\;Y_{c}\;Z_{c}\right]}^{T}$ the center of the ellipsoids. To fit the model to real humans, we set the average heights of children, juveniles and adults as 100 cm, 140 cm and 180 cm, respectively. The ratio of the head, torso and leg is set to 2:4:4.

**Figure 2:**
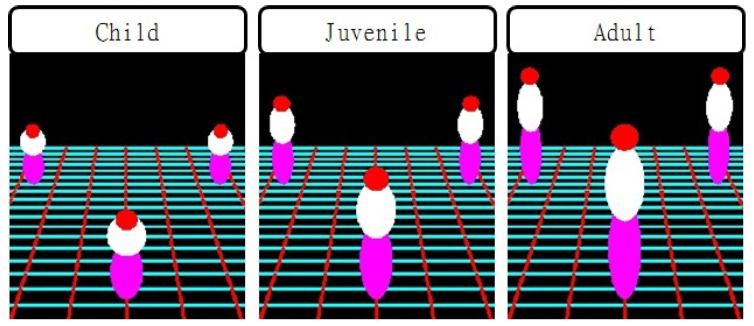
Human models on the projected multiple ellipses with different sizes and locations.

Each ellipsoid is back-projected to match a real object in the 2D space. The back-projected 3×3 ellipse, denoted as $C_{k}$, by projection matrix *P* is define as:

(3) $$C_{k}^{-1}=PQ_{k}^{-1}P^{T}$$

where *C* represents the ellipsoid matrix, such as $u^{T}Cu=0$. Figure 2 shows the result of the back-projected multiple ellipsoids at different positions. In each dotted box, three different ellipsoids have the same height.

The multiple ellipsoid-based human model is generated according to the position and height of an object from multiple cameras. The first step of generating the human model is to perform shape matching in the image. To match the shape, the proposed algorithm detects a moving object region by modeling the background using the Gaussian mixture model (GMM) [14] and then normalizes the detected shape. Since the apparent shape differs by the location and size of the object, the normalized shape is represented by a set of boundary points. More specifically, each boundary point is generated where a radial line from the center of gravity meets the outmost boundary of the object. If the angle between adjacent radial lines is *θ*, the number of boundary points is $N=360^{\circ }/\theta$. The shapes of an object and the corresponding human model are respectively defined as:

(4) $$B=\left[\begin{array}{cccc} j_{1} & j_{2} & \cdots & j_{N} \end{array}\right],\;\text{and}\;M_{i}=\left[\begin{array}{cccc} o_{1}^{i} & o_{2}^{i} & \ldots & o_{N}^{i} \end{array}\right]$$

where *B* represents the shape of the object, i∈{children,juvenile,adult}, $M_{i}$ the shape of the human model and *N* the number of normalized shapes. In this work, we experimentally used $\theta =5^{\circ }$, which results in N=72. The matching error between *B* and $M_{i}$ is defined as:

(5) $$e_{i}=\sum_{l=1}^{N}{\left(j_{l}-o_{l}^{i}\right)}^{2}$$

**Figure 3:**
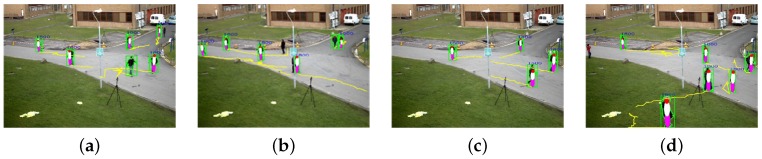
Matching results of the human models: (**a**) an example of the fitting failure in the second human from the right; (**b**–**d**) the corrected fitting results.

As a result, we select an ellipsoid-based human model with the minimum matching error $e_{i}$ to three human models, including child, juvenile and adult. If the matching error is greater than a threshold $T_{e}$, the object is classified as nonhuman. If the threshold $T_{e}$ is too big, nonhuman objects are classified as human. On the other hand, very small $T_{e}$ makes human detection fail. For that reason, we chose $T_{e}=8$ for the experimentally best human detection performance. The shape matching results of the ellipsoid-based human model appropriately fit real objects, as shown in Figure 3, where moving pedestrians are detected and fitted by the ellipsoid-based human model. The ellipsoid-based fitting fails when a moving object is erroneously detected. However, the rest of the correct fitting results can compensate for the occasional failure.

## 3. Human Model-Based Automatic Scene Calibration

Cameras with different internal and external parameters produce different sizes and velocities in the 2D image plane for the same object in the 3D space. In order to identify the same object in a multiple camera-based surveillance system, detection and tracking should be performed in the 3D world coordinate that is not affected by camera parameters. Normalized physical information of an object can be extracted in two steps: (i) automatic scene calibration to estimate the projective matrix of a camera [15,16,17]; and (ii) projection of the object into the world coordinate using the projective matrix. The proposed automatic calibration algorithm assumes that the foot-to-head line of a human object is orthogonal to the xy plane and parallel to the *z*-axis in the world coordinate.

The proposed human model-based automatic scene calibration consists of three steps: (i) extraction of foot and head candidate data to compute foot-to-head homology; (ii) homology estimation using foot-to-head inlier data; and (iii) camera calibration by estimating vanishing points and lines using the foot-to-head homology.

### 3.1. Foot-To-Head Homology

**Figure 4:**
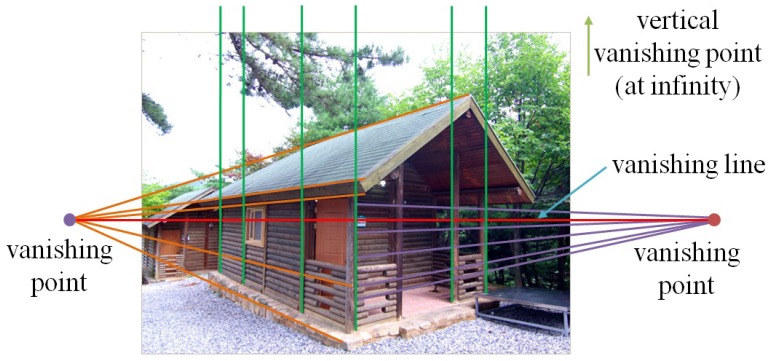
Vanishing lines and vanishing points.

In the Euclidean geometry, two parallel lines do not meet anywhere. On the other hand, in the projective geometry, two parallel lines meet at a point called the vanishing point. A line connecting two vanishing points is called the vanishing line, as shown in Figure 4.

**Figure 5:**
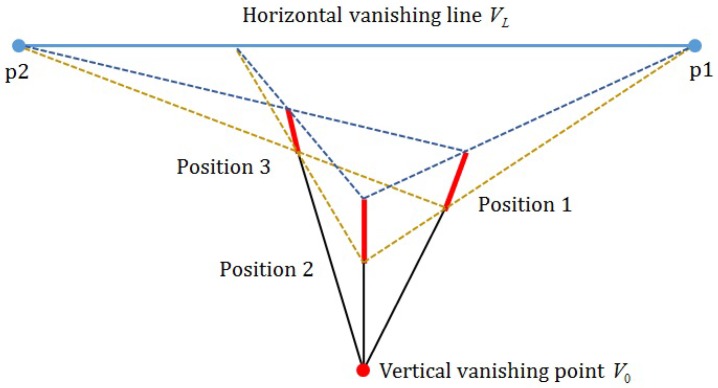
Estimation of vanishing lines and vanishing points.

Existing single image-based methods to estimate vanishing points and lines often fail if there are no line components in the background image [18,19]. In order to overcome the limit of background generation-based methods, a foreground object-based vanishing point detection method was recently proposed [15,16,17]. Since a general surveillance system has a camera installed at a higher position than the ground to view down objects, foot-to-head lines of a standing person at various positions on the ground, which is equivalent to the XY plane in the world coordinate, converge to a single point below the ground plane, as shown in Figure 5, where each position of the person is represented by a line segment with the bottom foot and the top head points. Extended foot-to-head lines meet at the vertical vanishing point $V_{0}$ below the ground level. The line connecting head points of Positions 1 and 2 meets another line connecting foot points of the same positions at p1. Likewise, p2 is determined by Positions 1 and 3. Based on the observation, three non-collinear positions of the person determine the horizontal vanishing line $V_{L}$ and the vertical vanishing point $V_{0}$.

The vanishing line and point are used to estimate the camera projection matrix. More specifically, let $\bar{X}={\left[X\;Y\;Z\;1\right]}^{T}$ be a point in the homogeneous world coordinate; its projective transformation becomes $\bar{x}=P\bar{X}$, where *P* is the projection matrix. Given $\bar{x}={\left[\bar{x}\;\bar{y}\;z\;1\right]}^{T}$, the corresponding point in the image plane is determined as $x=\bar{x}/z$, and $y=\bar{y}/z$. Since we assume that the XY plane is the ground plane, the foot position in the world coordinate is $X_{f}={\left[X\;Y\;0\right]}^{T}$ and the projected foot position is ${\bar{x}}_{f}=H_{f}{\bar{X}}_{f}$, where ${\bar{X}}_{f}={\left[X\;Y\;Z\;1\right]}^{T}$. In the same manner with the XY plane moving to the head plane, we have ${\bar{x}}_{h}=H_{h}{\bar{X}}_{h}$, where both $H_{f}$ and $H_{f}$ are 3×3 matrices. Since a head position is projected onto the corresponding foot position, such as ${\bar{X}}_{f}={\bar{X}}_{h}$,

(6) $${\bar{x}}_{h}=H_{hf}{\bar{x}}_{f},\;\text{and}\;{\bar{x}}_{f}=H_{fh}{\bar{x}}_{h}$$

where both $H_{hf}=H_{h}H_{f}^{-1}$ and $H_{fh}=H_{f}H_{h}^{-1}$ are 3×3 matrices and $H_{hf}=H_{fh}^{-1}$. Given the coordinate of a foot position in the ground plane, the corresponding head position in the image plane can be determined using $H_{hf}$. $H=H_{fh}$ is defined as the foot-to-head homology, and can be determined by computing the projection matrix *P* using the vanishing point, vanishing line and the object height *Z*.

### 3.2. Automatic Scene Calibration

The automatic scene calibration process consists of three steps: (i) extraction of foot and head inlier data; (ii) estimation of foot-to-head homology using the extracted inlier data; and (iii) detection of vanishing line and points. For the first step of the scene calibration, a human object is detected using the Gaussian mixture model. The detected object region goes through a morphological operation for noise-free labeling [20]. The inlier candidate of the foot and head of the labeled object is selected on two conditions: (i) a foot-to-head line should be inside a finite region with respect to the *y*-axis; and (ii) the foot-to-head line should be a major axis of an ellipsoid that will approximate the human object.

In order to obtain the angle, major axis and minor axis of the labeled human object, ellipse fitting is performed. More specifically, the object shape is defined by the external boundary as:

(7) $$S={\left[\begin{array}{cccc} s_{1} & s_{2} & \ldots & s_{N} \end{array}\right]}^{T}$$

where $s_{i}={\left[x_{i}\;y_{i}\right]}^{T}$, for i=1,…,N, represents the *i*-th boundary point and *N* the number of total boundary points. Using the second moments [21], the angle of shape *S* is computed as:

(8) $$\theta =\frac{1}{2}\arctan\left(\frac{2\mu_{1,1}}{\mu_{2,0}-\mu_{0,2}}\right)$$

where:

(9) $$\mu_{p,q}=\sum_{i=1}^{N}{\left(x_{i}-x_{c}\right)}^{p}{\left(y_{i}-y_{c}\right)}^{q}$$

and:

(10) $$x_{c}=\frac{1}{N}\sum_{i=1}^{N}x_{i},\;\text{and}\;y_{c}=\frac{1}{N}\sum_{i=1}^{N}y_{i}$$

In order to compute the major and minor axes of the ellipsoid, we first define the minimum and maximum inertial moments respectively as:

(11) $$\begin{array}{c} \begin{array}{c} I_{\min}=\sum_{i=1}^{N}\left\{\left(x_{i}-x_{c}\right)\cos\theta -\left(y_{i}-y_{c}\right)\sin\theta \right\}\\ I_{\max}=\sum_{i=1}^{N}\left\{\left(x_{i}-x_{c}\right)\sin\theta -\left(y_{i}-y_{c}\right)\cos\theta \right\} \end{array} \end{array}$$

The major and minor axes are determined using $I_{\min}$ and $I_{\max}$ as:

(12) $$A_{l}={\left(\frac{4}{\pi }\right)}^{1/4}{\left(\frac{I_{\max}^{3}}{I_{\min}}\right)}^{1/8},\;\text{and}\;A_{s}={\left(\frac{4}{\pi }\right)}^{1/4}{\left(\frac{I_{\min}^{3}}{I_{\max}}\right)}^{1/8}$$

The aspect ratio of the object is defined as $r=A_{l}/A_{s}$, and a candidate foot and head vector is defined as $c={\left[x_{f}\;y_{f}\;x_{h}\;y_{h}\right]}^{T}$. *c* is computed using *θ* as:

(13) $$\begin{array}{c} \begin{array}{c} x_{f}=\left(y_{\max}-y_{c}\right)\frac{\cos\theta }{\sin\theta }+x_{c},\mathrm{and}y_{f}=y_{\max}\\ x_{h}=\left(y_{\min}-y_{c}\right)\frac{\cos\theta }{\sin\theta }+x_{c},\mathrm{and}y_{h}=y_{\min} \end{array} \end{array}$$

where $y_{\max}$ and $y_{\min}$ respectively represent the maximum and minimum of $y_{i}$, for i=1,…,N.

The set of inlier candidates $C={\left[c_{1}\;c_{2}\;\cdots \;c_{L}\right]}^{T}$ is generated from $c_{i}$’s that satisfy four conditions: (i) $r_{1}<r<r_{2}$; (ii) $\theta_{1}<\theta <\theta_{2}$; (iii) there exist $s_{i}$ whose distance from $\left(x_{f},y_{f}\right)$ is smaller than $d_{1}$, and $s_{j}$ whose distance from $\left(x_{h},y_{h}\right)$ is smaller than $d_{1}$; and (iv) there are no pairs of $c_{i}$’s whose distance is smaller than $d_{2}$. In the first condition, $r_{1}=2$ and $r_{2}=5$ are used, and in the second condition, $\theta_{1}=80^{\circ }$ and $\theta_{2}=100^{\circ }$ are used for the experimentally best result. In the third and fourth conditions, $d_{1}=3$ and $d_{2}=10$ are respectively used.

Since the inlier candidate *C* still contains outliers, a direct computation of foot-to-head homology *H* results in a significant error. To solve this problem, we remove outliers in *c* using a robust random sample consensus (RANSAC) algorithm [22]. *H* can be determined using four inlier data since its degree of freedom is eight. Let $a={\left[h_{11}\;h_{12}\;h_{13}\;h_{21}\;h_{22}\;h_{23}\;h_{31}\;h_{32}\right]}^{T}$ be a vector whose eight elements are the first, row-ordered eight components of *H*; then, *a* can be determined by solving:

(14) $$\left[\begin{array}{cccccccc} x_{f} & y_{f} & 1 & 0 & 0 & 0 & -x_{f}x_{h} & -y_{f}y_{h}\\ 0 & 0 & 0 & x_{f} & y_{f} & 1 & -x_{f}y_{h} & -y_{f}y_{h} \end{array}\right]a=\left[\begin{array}{c} x_{h}\\ y_{h} \end{array}\right]$$

Since Equation (14) generates two linear equations given a candidate vector, four candidate vectors can determine *H*. In order to check how many inlier data support the estimated *H*, the head position of each candidate vector is estimated using *H*, which is determined by the corresponding foot position. The estimated head position is compared to the real head position, and the candidate vector is considered to support *H* if the error is sufficiently small. This process repeats a given number of times, and candidate vectors that support the optimal *H* become inliers. The inliers generate Equation (14). Since many inliers generally produce more than eight equations, vector *a*, which is equivalent to matrix *H*, is finally determined using the pseudo inverse. Although outliers can be generated by occlusion, grouping and non-human objects, the correct inlier data can be estimated while the process repeats and candidate data are accumulated.

Given the estimated foot-to-head homology *H*, arbitrarily chosen two foot positions generate corresponding two head positions. Two lines connecting the two pairs of feet and head positions meet at the vanishing point. More specifically, a line in the 3D coordinate can be represented using a vector $l={\left[a\;b\;c\right]}^{T}$, which satisfies the linear equation:

(15) ax+by+c=0

where the line coefficients {a,b,c} are determined using two points $p={\left[p_{x}\;p_{y}\right]}^{T}$ and $q={\left[q_{x}\;q_{y}\right]}^{T}$ as:

(16) $$\begin{array}{ccc} a & = & p_{y}-q_{y}\\ b & = & p_{x}-q_{x}\\ c & = & \left(p_{y}-q_{y}\right)q_{x}+\left(p_{x}-q_{x}\right)q_{y} \end{array}$$

If two lines $l_{1}$ and $l_{2}$ meet at the vanishing point $V_{0}$, the following relationship is satisfied:

(17) $$V_{0}=l_{1}\times l_{2}$$

**Figure 6:**
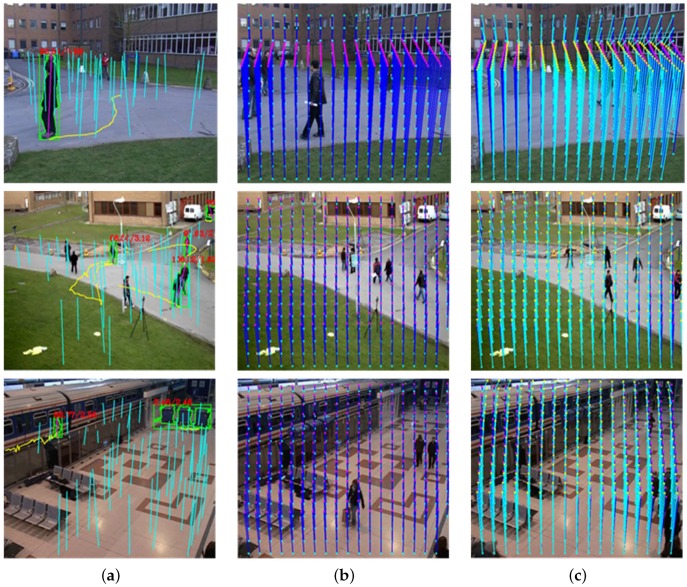
Foot-to-head homology estimation: (**a**) inlier data; (**b**) ground truth of the homology; and (**c**) the estimated homology.

In order to determine the vanishing line, three candidate vectors $\left\{c_{1},c_{2},c_{3}\right\}$ are needed. Two lines connecting both feet and head pairs connecting $c_{1}$ and $c_{2}$ meet at a point, say $r={\left[r_{x}\;r_{y}\right]}^{T}$. Likewise, another point $s={\left[s_{x}\;s_{y}\right]}^{T}$ is determined using $c_{2}$ and $c_{3}$. The line connecting two points *r* and *s* is the vanishing line $V_{L}$. Given $V_{0}$ and $V_{L}$, camera parameters can be estimated as shown in Figure 6.

### 3.3. Camera Parameter Estimation

Internal parameters include focal length *f*, principal point ${\left[c_{x}\;c_{y}\right]}^{T}$ and aspect ratio *a*. Assuming that the principal point is equal to the image center, a=1, and there is no skew, the simplified internal camera parameters are given as:

(18) $$K=\left[\begin{array}{ccc} f & 0 & c_{x}\\ 0 & f & c_{y}\\ 0 & 0 & 1 \end{array}\right]$$

External parameters include panning angle *α*, tilting angle *θ*, rolling angle *ρ*, camera height with respect to the *z*-axis and translations in the *x* and *y* directions. Assuming that α=0, x=y=0, the camera projection matrix is obtained by the multiplication of the internal and external parameter matrices as:

(19) $$P=K\left[\begin{array}{ccc} \cos\rho & -\sin\rho & 0\\ \sin\rho & \cos\rho & 0\\ 0 & 0 & 1 \end{array}\right]\left[\begin{array}{ccc} 1 & 0 & 0\\ 0 & \cos\rho & -\sin\rho \\ 0 & \sin\rho & \cos\rho \end{array}\right]\left[\begin{array}{cccc} 1 & 0 & 0 & 0\\ 0 & 1 & 0 & 0\\ 0 & 0 & 1 & -h_{c} \end{array}\right]$$

The vertical vanishing point with respect to the *z*-axis $v_{0}={\left[v_{x}\;v_{y}\;1\right]}^{T}$ provides the following constraint together with a point ${\left[x\;y\;1\right]}^{T}$ on the horizontal vanishing line:

(20) $$v_{0}^{T}\omega \left[\begin{array}{c} x\\ y\\ 1 \end{array}\right]=0$$

where $w=K^{-T}K^{-1}$ represents the image of the absolute conic (IAC). Substitution of Equation (18) into Equation (20) yields [23]:

(21) $$v_{x}x+\frac{v_{y}}{a^{2}}+f^{2}=0$$

which demonstrates that the horizontal vanishing line can be determined by the vertical vanishing point and the focal length and that rotation parameters can be computed from $v_{x}$, $v_{y}$, *f* as [8]:

(22) $$\rho =\arctan\frac{-av_{x}}{v_{y}},\mathrm{and}\theta =\arctan2\left(\sqrt{a^{2}v_{x}^{2}+v_{y}^{2}}-af\right)$$

where a=1.

The proposed algorithm can compute *f*, *ρ* and *θ* by estimating the vanishing line and point using Equations (21) and (22). The camera height $h_{c}$ can be computed using the real height of an object in the world coordinate $h^{w}$, vanishing line $v_{L}$ and vanishing point $v_{0}$:

(23) $$\frac{h^{w}}{h_{c}}=1-\frac{d\left(p_{h},V_{L}\right)d\left(p_{f},V_{0}\right)}{d\left(p_{f},V_{L}\right)d\left(p_{h},V_{0}\right)}$$

where $p_{f}$ and $p_{h}$ respectively represent the foot and head positions of the *i*-th object and d(a,b) the distance between points *a* and *b*. In the experiment, $h^{w}=180$ cm is used for the reference height.

## 4. Indexing of Object Characteristics

After object-based multiple camera calibration, the metadata of an object should be extracted given a query for the normalized object indexing. In this work, queries of an object consist of a representative color in the HSV color space, horizontal and vertical sizes in meters, moving speed in meters per second, the aspect ratio and moving trajectory.

### 4.1. Extraction of Representative Color

The color temperature of an object may change when a different camera is used. In order to minimize the color variation problem, the proposed work performs color constancy as a pre-processing step to compensate for the white balance of the extracted representative color.

#### 4.1.1. Color Constancy

If we assume that an object is illuminated by a single light source, the estimated color of the light source is given as:

(24) $$e=\left[\begin{array}{c} R_{e}\\ G_{e}\\ B_{e} \end{array}\right]=\int_{\omega }e\left(\lambda \right)s\left(\lambda \right)c\left(\lambda \right)d\lambda$$

where e(λ) represents the light source, s(λ) the reflection ratio of the surface, $c={\left[R\left(\lambda \right)\;G\left(\lambda \right)\;B\left(\lambda \right)\right]}^{T}$ the camera sensitivity function and *w* the wavelength spectrum, including the red, green and blue colors.

The proposed color compensation method is based on the shades of gray method [24,25]. The input image is down-sampled to reduce the computational complexity, and simple low pass filtering is performed to reduce the noise effect. The modified Minkowsky norm-based color with the consideration of local correlation is given as:

(25) $${\left(\frac{\int {\left(f^{\sigma }\left(x\right)\right)}^{p}dx}{\int dx}\right)}^{1/p}=ke$$

where f(x) represents the image defined on $x={\left[x\;y\right]}^{T}$, $f^{\sigma }=f*G^{\sigma }$ the filtered image by the Gaussian filter $G^{\sigma }$ and *p* the parameter of the Minkowski norm. A small *p* makes the uniform distribution of weights between measurement values, and vice versa. An appropriate choice of *p* prevents the light source from being biased to a specific color channel. In the experiment, p=6 was used for the experimentally best results for multiple camera color compensation. As a result, scaling parameters $\left\{w_{R},w_{G},w_{B}\right\}$ can be determined using the estimated color of the light source. The corrected color is given as:

(26) $$f_{\mathrm{corr}}^{c}=f_{c}/\omega_{c}^{3},\mathrm{for}c\in \left\{R,G,B\right\}$$

**Figure 7:**
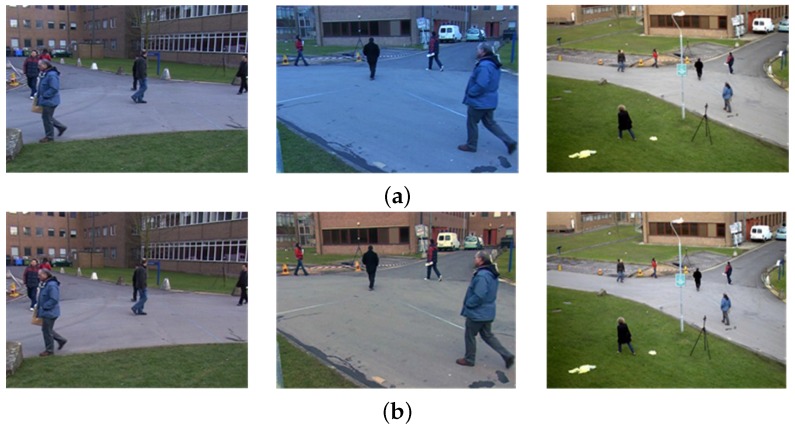
Results of color correction: (**a**) input images captured by three different cameras; and (**b**) color-corrected images using the shades of gray method.

Figure 7 shows the results of color correction using three different cameras. Color correction can also minimize the inter-frame color distortion, since it estimates the normalized light source.

#### 4.1.2. Representative Color Extraction

The proposed color extraction method uses the K-means clustering algorithm. An input RGB image is transformed to the HSV color space to minimize the inter-channel correlation as:

(27) $$H=\arctan\frac{\sqrt{3}\left(G-B\right)}{\left(R-G)+(R-B\right)},\;S=1-\frac{\min(R,G,B)}{V},\;V=\frac{R+G+B}{3}$$

Let $j_{n}={\left[H_{n}\;S_{n}\;V_{n}\right]}^{T}$ be the HSV color vector of the *n*-th pixel, for n=1,…,N, where *N* is the total number of pixels in the image. Initial *K* pixels are arbitrarily chosen to make a set of mean vectors $\left\{g_{1}\;\cdots \;g_{K}\right\}$, where $g_{i}$, for i=1,…,K, represents the selected HSV color vector. For every color vector, if $j_{n}$ is the closest to $g_{i}$, $j_{n}$ has the label $J_{i}$ as:

(28) $$J_{i}=\left\{j_{n}|d\left(j_{n},g_{i}\right)\leq d\left(j_{n},g_{b}\right),\mathrm{for}\;b=1,\cdots ,K\right\}$$

**Figure 8:**
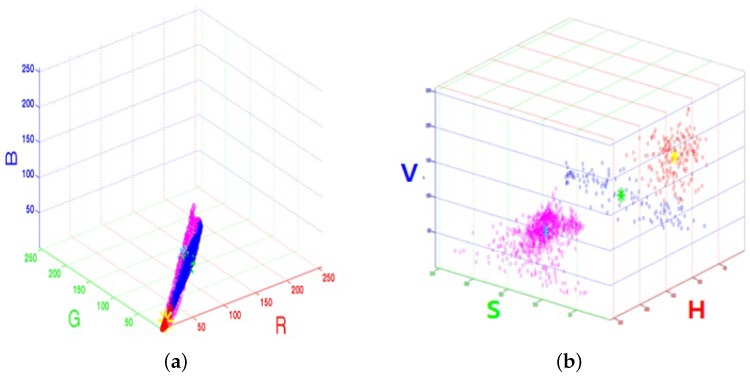
K-means clustering results in the (**a**) RGB and (**b**) HSV color spaces.

Each mean vector $g_{i}$ is updated by the mean of $j_{n}$’s in the cluster $J_{i}$, and the entire process repeats until there are no more changes in $g_{i}$. Figure 8 shows the results of K-means clustering in the RGB and HSV color spaces with K=3.

**Figure 9:**
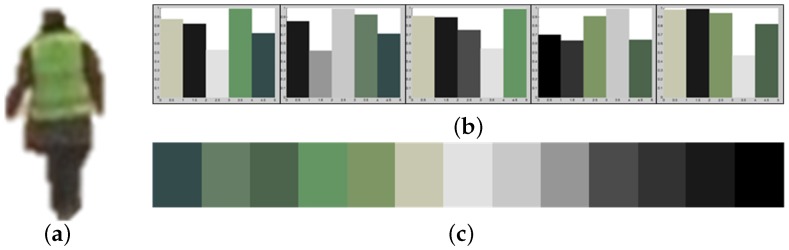
Results of K-means clustering to extract representative colors of the same object using different sets of initial clusters: (**a**) input image; (**b**) different results of K-means clustering; and (**c**) the sorted colors of (b).

**Figure 10:**
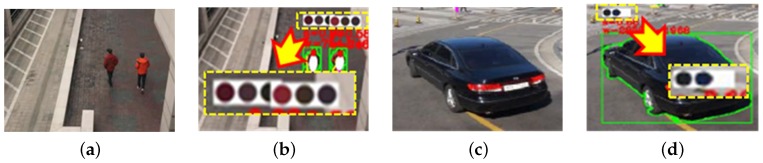
Selection of the representative colors from the candidate colors computed by the K-means clustering algorithm: (**a**) input image with two people; (**b**) the result of color selection; (**c**) an input image with a vehicle; (**d**) the result of color selection.

The fundamental problem of the K-means clustering algorithm is the dependency on the initial set of clusters, as shown in Figure 9. Since a single try of K-means clustering cannot guarantee extracting the representative colors, each frame generates candidate colors while tracking an object, and only the top 25% colors in the sorted candidates are finally selected. As a result, the representative colors of the object are correctly extracted even with a few errors. Figure 10 shows objects with extracted representative colors.

### 4.2. Non-Color Metadata: Size, Speed, Aspect Ratio and Trajectory

When multiple cameras are used in a video surveillance system, object size and speed are differently measured by different cameras. In order to extract the normalized metadata of an object, physical object information should be extracted in the world coordinate using accurately-estimated camera parameters.

#### 4.2.1. Normalized Object Size and Speed

We can compute the physical object height in meters if the projection matrix *P* and foot and head coordinates are in the image plane. In order to extract the physical information of an object in the world coordinate, the foot position on the ground plane ${\tilde{X}}_{f}=H^{-1}{\tilde{x}}_{f}$ should be computed using Equation (1). On the other hand, the *y* coordinate in the image plane is computed as:

(29) $$y=\frac{P_{2,1}\cdot X+P_{2,2}\cdot Y+P_{2,3}\cdot H_{o}+P_{2,4}}{P_{3,1}\cdot X+P_{3,2}\cdot Y+P_{3,3}\cdot H_{o}+P_{3,4}}$$

where *P* represents the projection matrix and $H_{0}$ the object height. Using Equation (29), $H_{0}$ can be computed from *y* as:

(30) $$H_{o}=\frac{\left(P_{2,1}-P_{3,1}\cdot y\right)X+\left(P_{2,2}-P_{3,2}\cdot y\right)Y+P_{2,2}-P_{3,2}\cdot y}{P_{3,3}\cdot y-P_{2,3}}$$

**Figure 11:**
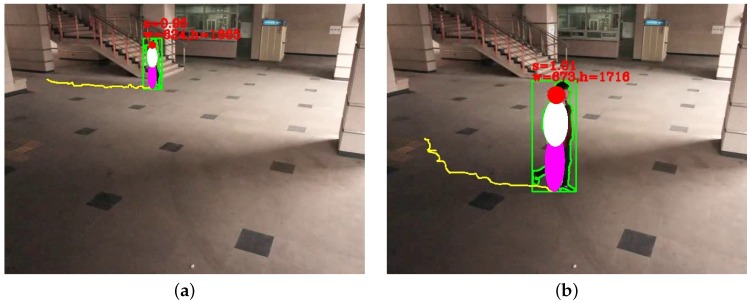
Size estimation results of the same object that is (**a**) far from the camera; (**b**) close to the camera.

The width of an object $W_{0}$ is computed as:

(31) $$W_{o}=\left|X_{o}-{X^{\prime }}_{o}\right|\cdot W_{i}$$

where $X_{0}$ represents the foot position in the world coordinate, $X_{0}^{\prime }$ the foot position that corresponds to the one-pixel shifted foot position in the image plane and $W_{i}$ the object width in the image plane. Figure 11 shows the results of normalized object size estimation. As shown in the figure, the estimated object height does not change while the object is moving around.

**Figure 12:**
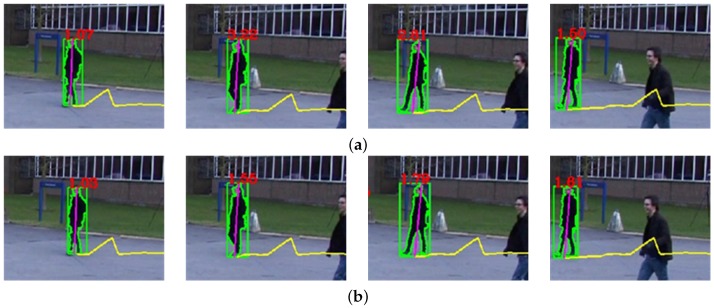
Results of object speed estimation: (**a**) without using the Kalman filter and (**b**) using the Kalman filter.

The object speed $S_{0}$ can be computed as:

(32) $$S_{o}=\sqrt{{\left(X_{o}^{t}-X_{o}^{t^{\prime }}\right)}^{2}+{\left(Y_{o}^{t}-Y_{o}^{t^{\prime }}\right)}^{2}}$$

where $\left(X_{0}^{t},Y_{0}^{t}\right)$ represents the object position in the world coordinate at the *t*-th frame and $\left(X_{0}^{t^{\prime }},Y_{0}^{t^{\prime }}\right)$ the previous object position by one second. However, the direct estimation of $S_{0}$ from the object foot position is not robust because of the object detection error. To solve the problem, the Kalman filter can compensate for the speed estimation error. Figure 12 shows the result of the object speed estimation with and without using the Kalman filter.

#### 4.2.2. Aspect Ratio and Trajectory

The aspect ratio of an object is simply computed as:

(33) $$R_{o}=H_{i}/W_{i}$$

where $H_{i}$ and $W_{i}$ respectively represent the object height and width in the image plane.

Instead of saving the entire trajectory of an object, the proposed system extracts object information using four positions in the trajectory. The object trajectory is defined as:

(34) $$T_{o}={\left[x_{o}^{1},y_{o}^{1},x_{o}^{2},y_{o}^{2},x_{o}^{3},y_{o}^{3},x_{o}^{4},y_{o}^{4}\right]}^{T}$$

where ${\left[x_{0}\;y_{0}\right]}^{T}$ is the starting position, ${\left[x_{1}\;y_{1}\right]}^{T}$ the 1/3 position, ${\left[x_{2}\;y_{2}\right]}^{T}$ the 2/3 position and ${\left[x_{4}\;y_{4}\right]}^{T}$ the ending position.

### 4.3. Unified Model of Metadata

Five types of metadata described in Section 4.1 and Section 4.2 should be unified into a single data model to be saved in the database. Since object data are extracted at each frame, median values of size, aspect ratio and speed data are saved at the frame right before the object disappears. Three representative colors are also extracted using the K-means clustering algorithm with the previously-selected set of colors.

The object metadata model, including object features, serial number and frame information, is shown in Table 1. As shown in the table, duration, moving distance and area size are used to sort various objects. For the future extension, minimum and maximum values of object features are also saved in the metadata.

## 5. Experimental Results

This section summarizes the experimental results of the proposed object-based automatic scene calibration and metadata generation algorithms. To evaluate the performance of the scene calibration algorithm, Table 2 summarizes the variation of object mean values captured in seven different scenes. The experiment extracts normalized physical information of a human object with a height of 175 cm in various scenes. As shown in Table 2, camera parameters were estimated and corrected at each scene. Object A appears 67 times, and object height is estimated every time.

**Figure 13:**
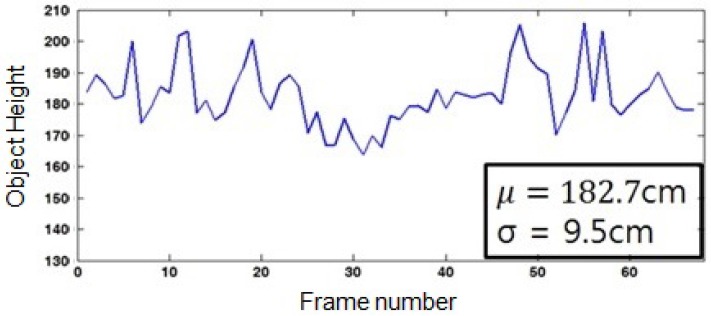
Variation of the object height in each frame.

Figure 13 shows that the average object height is 182.7 cm with a standard deviation 9.5 cm. Since the real height is 175 cm, the estimation error is 7.5 cm, because the reference height $h_{w}$ was set to 180 cm. This result reveals that the proposed calibration algorithm is suitable to estimate the relative height rather than the absolute value.

**Figure 14:**
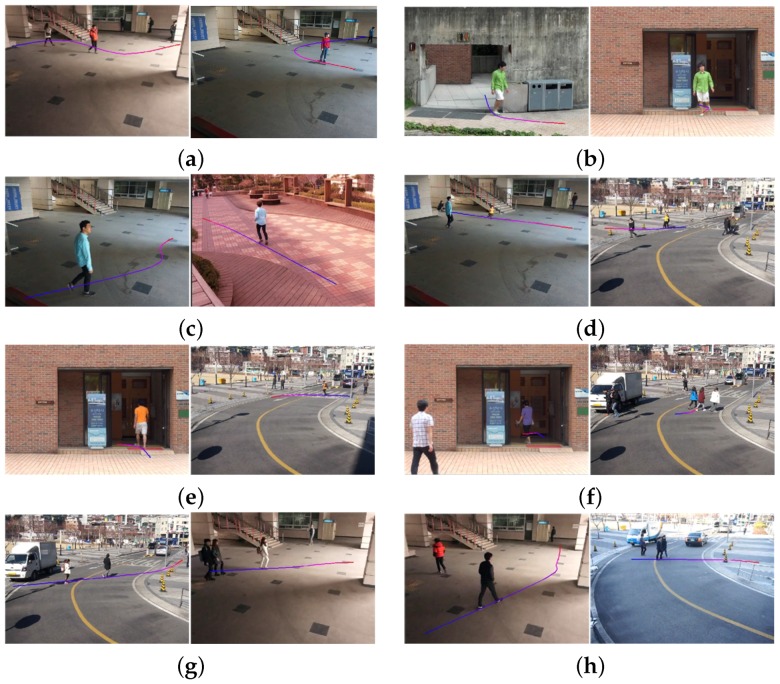
Results of the object search using representative colors. (**a**) Red; (**b**) green; (**c**) blue; (**d**) yellow; (**e**) orange; (**f**) purple; (**g**) white; (**h**) black.

Figure 14 shows the experimental results to search an object using the color query, including red, green, blue, yellow, orange, purple, pink, brown, white, gray and black. Table 3 summarizes the classification performance using the object color. The rightmost column has the number of total objects and the correctly classified ones in the parenthesis. The experiment can correctly classify 96.7% of the objects on average.

**Figure 15:**
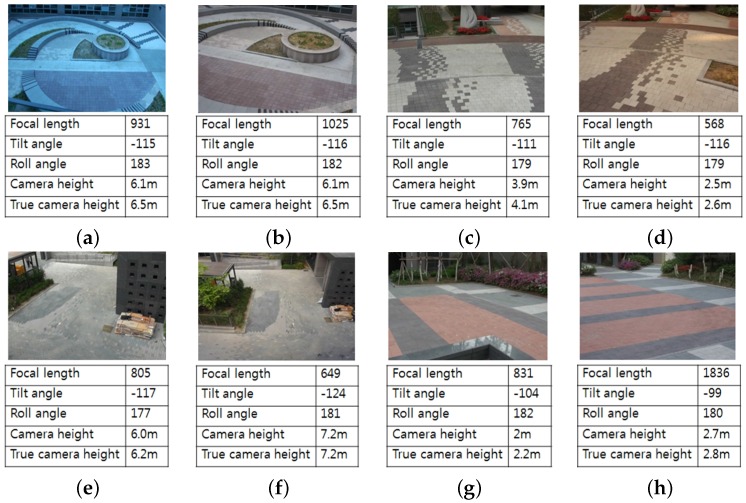
Test video files with estimated camera parameters: (**a**,**b**) two images of the first scene captured by two different camera parameters; (**c**,**d**) two images of the second scene captured by two different camera parameters; (**e**,**f**) two images of the third scene captured by two different camera parameters; (**g**,**h**) two images of the fourth scene captured by two different camera parameters.

**Figure 16:**
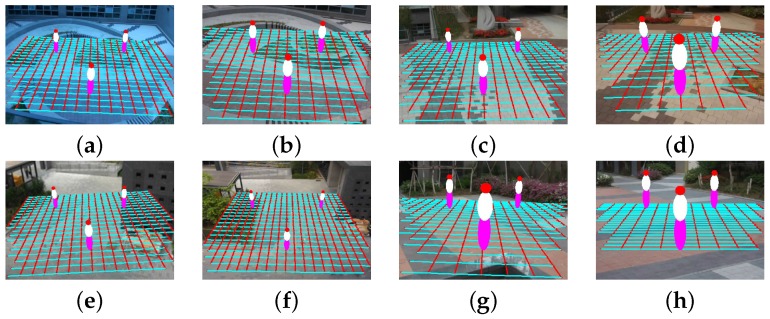
Result of camera calibration on the virtual three-dimensional grid on: (**a**,**b**) two images of the first scene captured by two different camera parameters; (**c**,**d**) two images of the second scene captured by two different camera parameters; (**e**,**f**) two images of the third scene captured by two different camera parameters; (**g**,**h**) two images of the fourth scene captured by two different camera parameters.

Figure 15 shows eight test videos with estimated camera parameters. Figure 16 shows the camera calibration results of eight test videos on the virtual ground plane and ellipsoids of a height of 180 cm.

**Figure 17:**
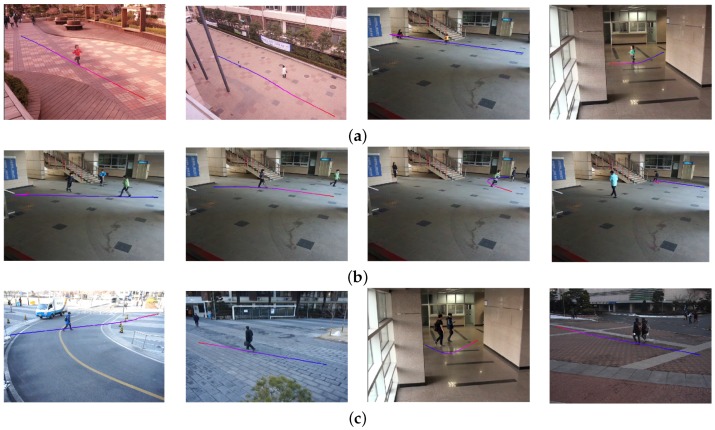
Results of the object using the object size. (**a**) Small size; (**b**) medium size; (**c**) large size.

Figure 17 shows the experimental results of the object search using the size query, including children (small), juveniles (medium) and adults (large). Figure 17a shows that the proposed algorithm successfully searched children smaller than 110 cm, and Figure 17b,c shows the similar results with a juvenile and adult, respectively. Table 4 summarizes the classification performance using the object size. The right most column has the number of total objects and the correctly-classified ones in the parenthesis. The experiment can correctly classify 95.4% of the objects on average.

**Figure 18:**
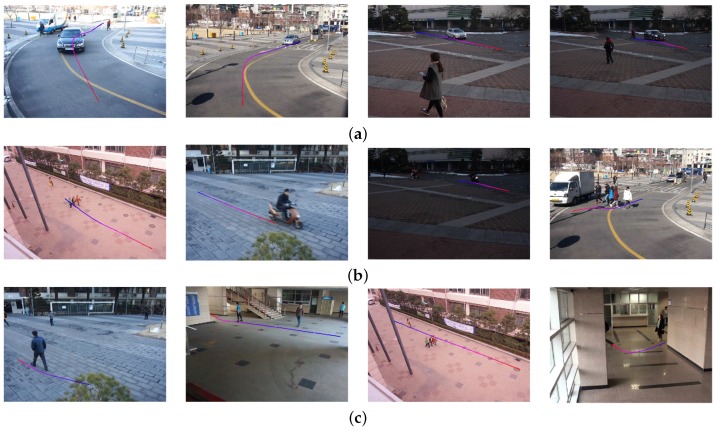
Results of the object search using the object ratio. (**a**) Horizontal; (**b**) normal; (**c**) vertical.

Figure 18 shows the experimental results of the object search using the aspect ratio. The horizontal query is used to find vehicles; the normal query is used to find motorcycles and groups of people; and the vertical query is used to find a single human object. Table 5 summarizes the classification performance using the aspect ratio. The rightmost column has the number of total objects and the correctly-classified ones in the parenthesis. The experiment can correctly classify 96.9% of the objects on average.

**Figure 19:**
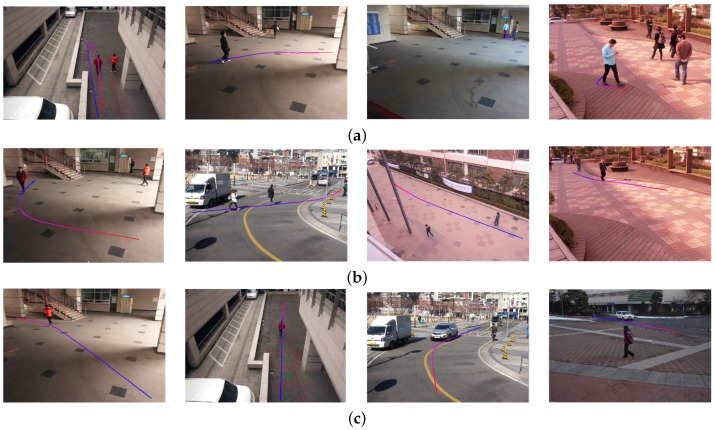
Results of the object search using the object speed. (**a**) Slow; (**b**) normal; (**c**) fast.

Figure 19 shows the experimental results of the object search using the speed queries, including slow, normal and fast. Table 6 summarizes the search results using the object speed with the classification performances. As shown in Table 6, more than 95% of the objects are correctly classified.

Table 3, Table 4, Table 5 and Table 6 show the accuracy and reliability of the proposed algorithm. More specifically, the color-based searching result shows relatively high accuracy with various searching options. For that reason, the object color can be the most important feature for object identification.

**Figure 20:**
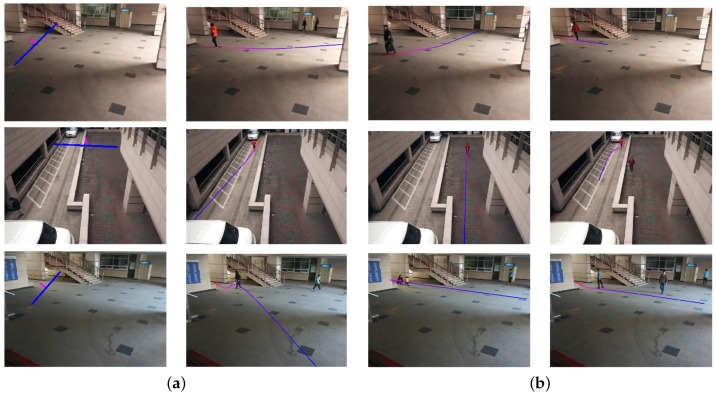
Results of the object search using the moving direction. (**a**) Line setting; (**b**) the results of the search.

Figure 20 shows the experimental results of the object search using user-defined boundaries to detect a moving direction.

**Figure 21:**
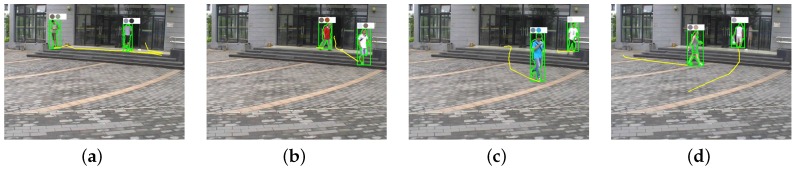
Results of the proposed algorithm using a public dataset [26]: (**a**–**d**) four frames in the test video with re-identified people.

Figure 21 shows the results of the proposed algorithm for person re-identification in the wild (PRW) dataset [26]. As shown in the figure, the objects’ colors and trajectories are correctly classified.

**Figure 22:**
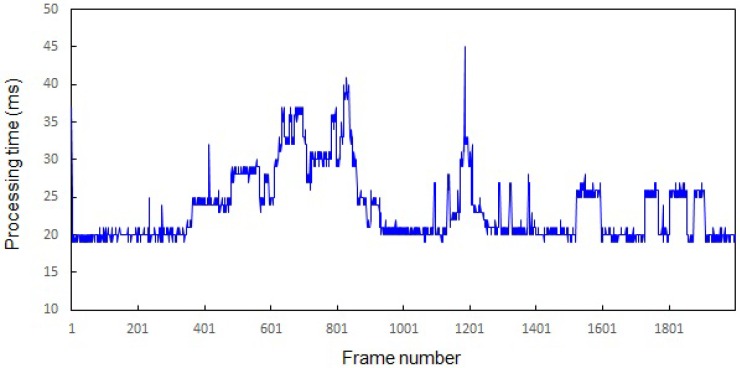
Processing time of the proposed algorithm.

Figure 22 shows the processing time of the proposed algorithm. To measure the processing time, a personal computer is used with a 3.6-GHz quad-core CPU and 8 GBytes of memory. As shown in Figure 22, it takes 20–45 ms to process a frame, and the average processing speed is 39 frames per second (FPS).

## 6. Conclusions

This paper presented a multiple camera-based wide-range surveillance system that can efficiently retrieve objects-of-interest by extracting normalized metadata of an object acquired by multiple, heterogeneous cameras. In order to retrieve a desired video clip from a huge amount of recorded video data, the proposed system allows a user to query various features, including the size, color, length ratio, moving speed and direction. The first step of the algorithm is the auto-calibration to extract normalized physical data. The proposed auto-calibration algorithm can estimate both the internal and external parameters of a camera without using a special pattern or depth information. Image data acquired by the appropriately-calibrated camera provides normalized object information. In the metadata generation step, a color constancy algorithm is first applied to the input image as preprocessing. After a set of representative colors are extracted using K-means clustering, the physical size and speed of an object-of-interest is estimated in the world coordinate using the camera parameters. The metadata of the object are then generated using the size ratio and motion trajectories. As a result, an object-of-interest can efficiently be retrieved using a query that combines physical information from big video data recorded by multiple, heterogeneous cameras. Experimental results demonstrated that the proposed system successfully extracts the metadata of the object-of-interest using three-dimensional (3D) human modeling and auto-calibration steps. The proposed method can be applied to a posteriori video analysis and retrieval systems, such as a vision-based central control system and a surveillance system.

## Figures and Tables

**Table 1:** Object metadata model.

Name		Description
ID		Object number
File name		Occurrence video file name
Frame	Start frame	Start frame, end frame and duration of the frame
	End frame	
	Duration	
Trajectory	First position	First position, 1/3 position, 2/3 position, last position and moving distance
	Second position	
	Third position	
	Last position	
	Moving distance	
Height (mm)	Min height	Minimum, median and maximum height of the object
	Median height	
	Max height	
Width (mm)	Min width	Minimum, median and maximum width of the object
	Median width	
	Max width	
Speed (m/s)	Min speed	Minimum, median and maximum speed of the object
	Median speed	
	Max speed	
Aspect ratio	Min aspect ratio	Minimum, median and maximum aspect ration of the object
	Median aspect ratio	
	Max aspect ratio	
Color	First color	First, second and third HSV color value
	Second color	
	Third color	
Area size	Min area	Minimum, median and maximum size of the area
	Median area	
	Max area	

**Table 2:** Performance evaluation of scene auto-calibration.

Input Scenes	Estimated and Corrected Camera Parameters	Scenes with A	Number of Appearances
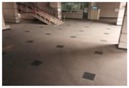 <Scene_1>	$\begin{array}{c} f=613\\ \theta =-111^{\circ }\\ \rho =182^{\circ }\\ h_{c}=2660\mathrm{mm} \end{array}$	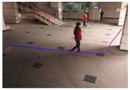	25
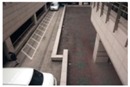 <Scene_2>	$\begin{array}{c} f=632\\ \theta =-118^{\circ }\\ \rho =180^{\circ }\\ h_{c}=6450\mathrm{mm} \end{array}$	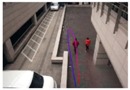	9
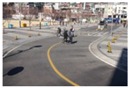 <Scene_3>	$\begin{array}{c} f=643\\ \theta =-104^{\circ }\\ \rho =180^{\circ }\\ h_{c}=3096\mathrm{mm} \end{array}$	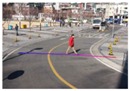	2
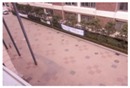 <Scene_4>	$\begin{array}{c} f=667\\ \theta =-117^{\circ }\\ \rho =173^{\circ }\\ h_{c}=10,331\mathrm{mm} \end{array}$	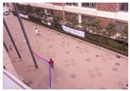	3
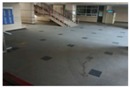 <Scene_5>	$\begin{array}{c} f=644\\ \theta =-107^{\circ }\\ \rho =183^{\circ }\\ h_{c}=2399\mathrm{mm} \end{array}$	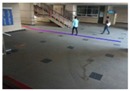	15
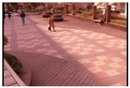 <Scene_6>	$\begin{array}{c} f=688\\ \theta =-108^{\circ }\\ \rho =179^{\circ }\\ h_{c}=2672\mathrm{mm} \end{array}$	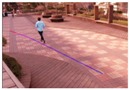	10
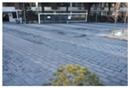 <Scene_7>	$\begin{array}{c} f=532\\ \theta =-109^{\circ }\\ \rho =180^{\circ }\\ h_{c}=3035\mathrm{mm} \end{array}$	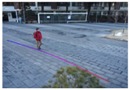	3

**Table 3:** Result of the classification based on the color.

	Red	Green	Blue	Yellow	Orange	Purple	Pink	White	Gray	Black	Total Object
Red	112	0	0	0	2	0	4	0	0	0	129 (95%)
Green	0	6	1	0	0	0	0	0	0	0	7 (86%)
Blue	0	1	96	0	0	0	0	0	4	3	104 (92%)
Yellow	0	0	0	7	0	0	0	1	0	0	8 (88%)
Orange	2	0	0	3	88	0	0	1	0	0	94 (94%)
Purple	0	0	0	0	0	2	0	0	0	0	2 (100%)
Pink	1	0	0	0	1	0	12	0	0	0	14 (86%)
White	0	0	0	0	0	0	0	79	5	0	84 (94%)
Gray	0	0	0	0	0	0	0	1	93	2	96 (97%)
Black	0	0	4	0	0	0	0	0	23	1237	129 (98%)

**Table 4:** Result of the classification based on the object size.

	Small	Medium	Large	Total Object
Small	35	11	3	49 (71%)
Medium	6	185	21	212 (87%)
Large	0	17	993	1010 (98%)

**Table 5:** Result of the classification based on the aspect ratio.

	Horizontal	Normal	Vertical	Total Object
Horizontal	38	3	5	46 (83%)
Normal	1	54	7	62 (87%)
Vertical	2	21	1140	1163 (98%)

**Table 6:** Result of the classification of the speed-based search.

	Slow	Normal	Fast	Total Object
Slow	96	37	0	133 (72%)
Normal	2	976	5	983 (99%)
Fast	0	9	146	155 (94%)

## Supplementary Materials

The following are available online at http://www.mdpi.com/1424-8220/16/7/963/s1.
